# Managing Emergency Situations in VANET Through Heterogeneous Technologies Cooperation

**DOI:** 10.3390/s18051461

**Published:** 2018-05-08

**Authors:** Amilcare Francesco Santamaria, Mauro Tropea, Peppino Fazio, Floriano De Rango

**Affiliations:** Dimes Department, University of Calabria, 87036 Rende, Cosenza, Italy; afsantamaria@dimes.unical.it (A.F.S.); mtropea@dimes.unical.it (M.T.); f.derango@dimes.unical.it (F.D.R.)

**Keywords:** Car-to-car Communications, VANET, WAVE, 802.11p, road safety, routing, vehicular, data dissemination, scalable architecture

## Abstract

Nowadays, the research on vehicular computing enhanced a very huge amount of services and protocols, aimed to vehicles security and comfort. The investigation of the IEEE802.11p, *Wireless Access in Vehicular Environments* (WAVE) and *Dedicated Short Range Communication* (DSRC) standards gave to the scientific world the chance to integrate new services, protocols, algorithms and devices inside vehicles. This opportunity attracted the attention of private/public organizations, which spent lot of resources and money to promote vehicular technologies. In this paper, the attention is focused on the design of a new approach for vehicular environments able to gather information during mobile node trips, for advising dangerous or emergency situations by exploiting on-board sensors. It is assumed that each vehicle has an integrated on-board unit composed of several sensors and *Global Position System* (GPS) device, able to spread alerting messages around the network, regarding warning and dangerous situations/conditions. On-board units, based on the standard communication protocols, share the collected information with the surrounding road-side units, while the sensing platform is able to recognize the environment that vehicles are passing through (obstacles, accidents, emergencies, dangerous situations, etc.). Finally, through the use of the GPS receiver, the exact location of the caught event is determined and spread along the network. In this way, if an accident occurs, the arriving cars will, probably, avoid delay and danger situations.

## 1. Introduction

In the first half of 2017, more than 80,000 road accidents with injuries to people happened only in Italy, causing more than 110,000 injuries, and 1600 deaths [1]. Every year in the world 1.25 million people die for road accidents an in one third of the cases the fault is the excessive speed. This is stated in a report published by the *World Health Organization* (WHO) [2]. Clearly, road accidents represent a big issue for the entire planet, due to people that lose their life on the roads. Given such dramatic numbers, from several years, the whole scientific community is continuously researching about new possibilities offered by the new technologies in vehicular environments. *Car-to-Car* (C2C) communication paradigm allows the development of many new applications in vehicular environment, especially related to the road safety. The increasing research and development activity in the *Intelligent Transportation System* (ITS) [3] environment is due to the huge diffusion of transport vehicles and, proportionally, to the number of accidents that happen. Generally, the main reason for these dramatic events is related to the violation of the traffic rules and to the bad driving styles: a solution that provides the cooperation of heterogeneous technologies (such as IEEE802.11p, GPS and Radar) could be helpful in preventing critical situations. The use of these standards and the related road infrastructures could help drivers to avoid accidents, introducing into the driving system a new set of information (infotainment) about road conditions, enhancing drivers’ sight along the road [4,5]. Recent researches try to exploit IEEE802.11p to allow multi-hop communications [6] and to make an efficient data dissemination [7,8], exploiting ad-hoc connections that could be created on the IEEE802.11p-based network. To prevent accidents when driving, current systems make use of vehicle detection through active (e.g., laser) or passive (e.g., camera) sensors. The laser allows to calculate the distance from other cars that come across during the trip. Passive sensors permit to acquire data in a non-intrusive way [9]. In [10] the authors show the importance of the beacon messages for exchanging vehicles information between them in order to create cooperative awareness and understanding the right frequency interval.

In this paper, in order manage in a scalable way car traffic, we propose a full integration framework between vehicle sensors and IEEE802.11p-based *On-Board Unit* (OBU) technology. Clearly, a communication protocol should be also properly designed, in order to deal with the data dissemination, maintaining the overhead under acceptable levels. The possibility to integrate the *Vehicular Ad-hoc Network* (VANET) components with vehicular equipments can improve safety and road management by collecting and analyzing data properly. In this work, the concept of OBU is extended in order to have the possibility to acquire data from vehicle’s sensors, to have a deeper knowledge of the surrounding environment. In this way, it is possible to act in a better way on protocol management by avoiding to flood unnecessary messages in the network. *Extended On-Board Unit* (E-OBUs) can exploit acquired data from sensors to know their neighbors and monitor them and, in case of strange behavior, it can act on the vehicle behavior to reduce damages or avoid collisions.

The main contributions of the paper are the following ones:

- A new layered architecture is proposed in order to manage dangerous situations (e.g., emergencies or accidents) in VANETs environments; in particular, it is designed an architecture composed of three layers: cloud layer (for global management), edge layer (for local and distributed management) and end system layer for heterogeneous technology integration;
- Some new messages for 802.11p have been defined to support the new scalable architectures;
- Some novel metrics have been considered in the VANET network assimilated to a graph structure;
- Several simulation campaigns have been extensively carried out to show the effectiveness of the architecture, protocols and adopted metrics in terms of traffic re-distribution and traveling time reduction.

The paper is organized as follows: in Section 2, the most important related works are discussed. Section 3 describes the proposed architecture, with its level and their functionalities, the proposed protocol with a deep analysis of the packet structure and timing diagram. Section 4 is dedicated to the description of the needed hardware, while Section 5 defines the graph related to the roads and lanes with the associated metrics. Finally, Section 6 describes the obtained simulation results and conclusions are summarized in Section 7.

## 2. Related Work

Many protocols and applications have been proposed in literature to deal with traffic physical security and comfort, especially after the incredible development of VANETs technologies. However, very few of them tend to integrate the available technologies, in order to exploit their advantages. For example, several applications have been developed to improve road safety by using GPS and users advisories: to reduce and optimize the time needed by an emergency vehicle, the accident detection is made by a direct connection with the car control unit, while calling directly the emergency number or sending data to a dedicated server [11]. HD traffic [12] is another solution developed by TomTom, allowing to receive up-to-date information related to the path the vehicle is following, collecting data from other devices that use this service. In [13], the authors provide an overview of an innovative approach for effective cross-network information dissemination (quick and inexpensive) of traffic alerts using VANET network architecture. In [14,15,16,17,18] the authors describe a new intelligent street lighting system integrated with VANET technology in the smart city context. The paper proposes a system able to tune the LED lamp automatically according to environment luminance changes. In [19] the authors develop a Traffic Management System (TMS) solution that incorporates the progress in the *Internet of Things* (IoT) technologies. The aim of [20] is to propose a route planning algorithm able to dynamically calculate the route that meets the shortest travelling time or the lowest fuel consumption criteria using information from Google Map. In [21] the authors proposed a dynamic traffic light control scheme, in order to reduce vehicles CO${}_{2}$ emissions. In the proposed scheme, vehicles can pass through intersections with less waiting time and fewer numbers of stops. Other works that propose solution for traffic lighting control based on traffic analysis are [22,23,24]. In [25] the authors address the road safety issue in VANET and by proposing a framework called *Attention Assist Framework* (AAF). In [26] the authors propose a detailed analysis on ITS that may help to improve actual solutions. The work in [27] deals with the *Adaptive Speed Limits* (ASLs) which is a technique for enhancing the driving conditions on the roads. The variable speed limit with notification in-vehicle driver is an important factor that can outperform dynamic roadside speed limit signs in terms of safety. The authors present a framework outlining the system-level design and the implementation for an ASL system with an interactive in-vehicle display supporting communication units. The authors in [28] presented a strategy to reduce traffic congestion using the *Vehicle-To-Vechicle* (V2V) communication. Periodically emitted beacons are used to analyze traffic flow and to warn other drivers of a possible distribution of traffic. The vehicles that receive such a warning, are told to keep a larger gap to their predecessor. In [29] the authors, on the basis of a hybrid ITS which utilizes both vehicular ad hoc networks and cellular systems, propose a real-time path-planning algorithm able to reduce average vehicle travel cost for avoiding congestion. The work in [30] presents a routing protocol that uses Real-Time traffic information gathered through the exchange of messages between vehicles. This protocol creates road-based paths consisting of successions of road intersections that have an high probability of connectivity among them. Another work that faces with safety issues is [31]: in this idea the vehicles collaborate in order to avoid collision. In [32,33,34], we have paid attention to the network layer of VANET, trying to reduce the co-channel interference during data exchange between the vehicles. Instead, in this paper we have focused the study on a new distributed architecture and a necessary protocol for managing dangerous situations in VANET networks.

## 3. Architecture and Communication Protocol

In this section, we provide a description of the considered model, based on a scalable network architecture. In order to better understand the main idea of this work we present a brief overview of the possible hardware solutions in the design of a customized OBU able to interface vehicle on-board sensors. In addition, a multilayer architecture is presented in order to address scalability issues, that may affect this kind of network [35]. Another challenge to be faced is the system reaction time to dangerous events such as car collisions or accidents that may involve ITS management tasks. In fact, commonly, ITSs are centralized and all tasks are performed in a specific node or exploiting cloud services. This approach may waste the overall ITS performances reducing its ability to solve congestion or to address emergencies. System responses may not reach end systems in time and some side effects such as delays in road assistance or first aids may happen. However, this approach is commonly used in ITS solution as proposed in [36]. In this work, instead, we propose a decentralized architecture at the edge able to distribute dynamically computational load towards local nodes approaching the Fog Computing [37] in the ITS and VANET environment [38].

### 3.1. Proposed Architecture

The proposed architecture is composed of three layers in order to make the system more scalable, efficient and able to respond faster to the dynamics that may happen in the real environment. This may help to reach our goal by reducing reaction time for humans and machines. The high level is composed of the *High Road Traffic Manager* (HRTM). It is instantiated on the cloud and cooperates with the *Low Road Traffic Manager* (LRTM), which is instantiated on edge layer. These two layers are responsible of coordinating traffic conditions by exploiting monitored data. These data are collected from observations on the field by exploiting *RoadSide Units* (RSUs), vehicles and road devices. In particular, HRTM brings information from LRTM and it elaborates them for achieving continuous data about road, area status and events that may change traffic load. These information are used by HRTM to optimize vehicle flows at global level by exploiting field devices such as traffic lights and smart road signs. In this way, HRTM can globally optimize traffic loads and reduce journey time. It may also dynamically change the city zone to *restricted* or *unrestricted* by allowing or not traffic flows in some areas. LRTM periodically communicates with local RSUs for collecting data about roads and traffic levels. LRTM is responsible of a direct interface with the covered RSUs by querying them or sending broadcasted alert messages in the network. It has other tasks to accomplish but they will be discussed deeply in the next sections. HRTM and LRTM are active actors in the optimizations of vehicle distribution inside the covered area. HRTM is responsible of the global optimization by dynamic rearranging weights that are used by vehicles to evaluate their paths. LRTM, instead, is responsible to local optimization and it communicates alerts when dangerous events occur. Both HRTM and LRTM exploit RSUs to disseminate information to reach vehicles.

**Figure 1:**
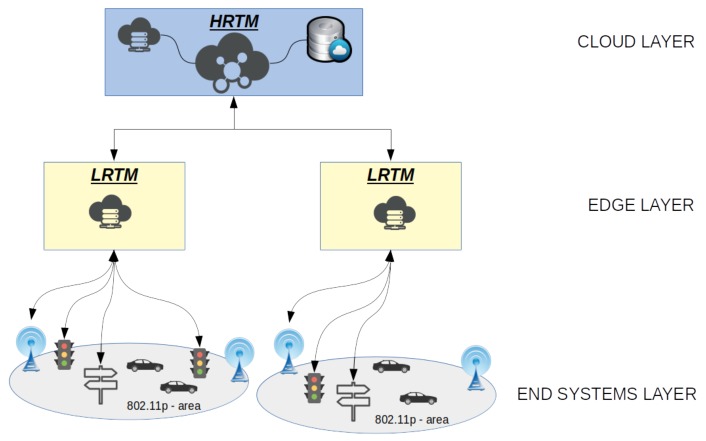
The proposed heterogeneous and scalable architecture used in *Vehicular Ad-hoc Network* (VANET) environment. Here three layers are responsible of the network management and services such as data dissemination, data collection, data storage and data elaboration.

In case of congestion or dangerous situation, each vehicle can re-evaluate its own itinerary based on new path computation with updated cost and constraints made by HRTM. The messaging system among vehicles and infrastructure is made exploiting the *Vehicle-To-Infrastructure* (V2I) link. Message format is based on the use of *Wave Short Message Protocol* (WSMP) [39,40,41], which allows *Vehicle-To-Everything* (V2X) communications [42]. This link is used by RSUs also to communicate the messaging coming from higher architecture level such as LRTM or HRTM. V2V communication link is also used by vehicles to disseminate information as happens in common VANETs architectures. Instead, regarding the Traffic lights and road signs they are controlled directly by LRTM exploiting wireless link by taking advantage of *Machine to Machine* (M2M) communication protocol to make more reliable and scalable networking, avoiding resource wastages. Regarding the core network, wired connections are used because of the delay constraints. In fact, it is important to have low delays, as well as possible, to make the system response time faster. As depicted in Figure 1 the end system layer is composed of heterogeneous devices.

#### 3.1.1. Cloud Layer

The cloud layer represents the core of the system and it is responsible of several global tasks. It acquires data from lower layers to analyze and update road conditions in a real-time way. This step allows ITS to improve the decision making process. The output is the global optimization of traffic distribution along available roads served by the system. Moreover, ITS is responsible to address emergencies that are reported from lower layers by arranging paths of rescue teams and allowing them to reach destinations in a short time.

#### 3.1.2. Edge Layer

**Figure 2:**
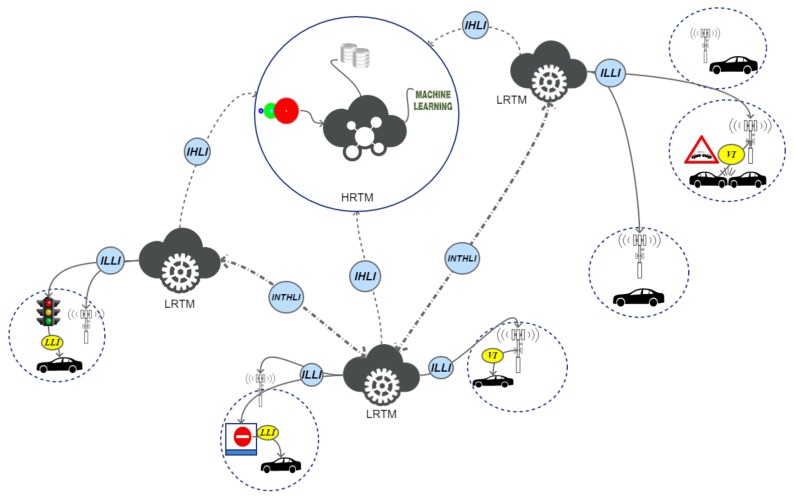
Interface integration in the heterogeneous architecture.

This layer is composed of several devices that have the main goal to accomplish local tasks such as communicate potentially dangerous events to vehicle, filtering collected data to transfer on cloud. Moreover, it provides a solution for local traffic distribution in case of emergency in order to avoid congestion on the involved area. In this way, the system is more flexible and it can react in a shorter time to events, dynamically rearrange traffic by sending ad-hoc messages to smart traffic lights and road signs. The LRTM is commonly an embedded device based on ARM architecture. It must be able to instantiate an embedded Linux distribution and to elaborate data coming from VANET layer. It has to accomplish several tasks related to the management of partial map areas. They can be placed with some RSU to extend their functionalities. It can communicate with several RSUs by exploiting dedicated interface called *Inter-Low-Layer Interface* (ILLI). It is depicted in Figure 2.

#### 3.1.3. End System Layer

End system layer is the most heterogeneous layer of the architecture because it is composed of two sub-layers:

- The vehicular layer is composed of VANET devices such as OBUs and RSUs. This layer is reachable by exploiting the capability of RSUs to communicate with other VANET devices, but it is also able to communicate with external networks. This layer is used to acquire data on traffic and road status by monitoring flows: data is then filtered and aggregated by LRTM and sent to HRTM;
- The traffic controller layer is composed of smart devices such as traffic lights and road signs that have been improved by equipping them with an elaboration unit. It allows devices to communicate with other layers (to actuate decisions made by LRTM or HRTM layers).

### 3.2. Protocol Communications

The overall system is based on the inter and intra communications of sub-layers. We design a hierarchical architecture for better distribute the computational load allowing faster response to those events that may be responsible of congestions. The presented architecture is based on three different layers that perform traffic management tasks. Each layer can communicate with its surrounding layers exploiting dedicated interfaces. Then, a communication protocol uses these interfaces for sending messages between layers. It has been designed to reduce the amount of data transferred and delays. It is based on the following interfaces:

***Inter-High-Layer Interface* (IHLI):** it defines rules for exchanging data between HRTM and LRTM layers;
***Intra-High-Layer Interface* (INTHLI):** This interface provides communication among LRTM systems;
***Inter-Low-Layer Interface* (ILLI):** it is used to exchange data between LRTM and RSUs, smart traffic lights and smart road signs;
***VANET Interface* (VI):** it is used by RSUs to communicate between them and to implement the V2V and V2I communication protocol;
***Low Layer Interface* (LLI):** it is used to allow communication between smart road elements and vehicles. These communications are useful for the whole system because of the vehicle flow monitoring.

These interfaces, which are summarized in Figure 2, allow inter- and intra-layers communication exploiting heterogeneous technologies.

**Figure 3:**
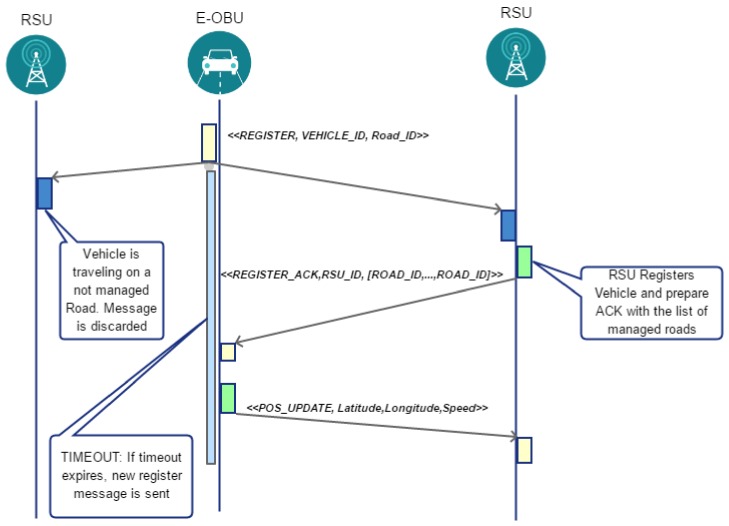
Protocol timing of *RoadSide Unit* (RSU) and E-OBU during the registering phase.

In this section the behavior of vehicles is described in detail. When a vehicle begins its journey, it evaluates through the analysis of the map the Road_ID. It sends a Register message in a broadcast way. Several RSU may receive this message but only the RSU that has the task to manage that Road_ID registers the vehicle in a local data structure and generates an Acknowledgment message. This message will bring up also the other Road_ID managed by the RSU. Thus, no more register messages will be generated by the car that is traveling on those roads. If no acknowledgment is received within a given timeout then another register message is sent. This mechanism is shown in a timing diagram depicted in Figure 3.

**Figure 4:**
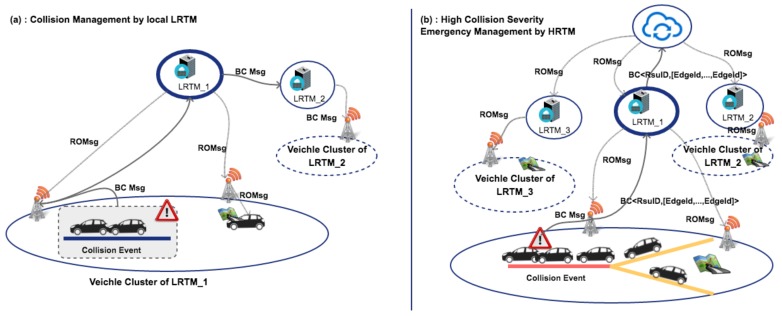
Collision management by sub-layers. Here vehicles and LRTM disseminate messages.

In case of an accident (e.g., collision between cars), we have designed the following protocol rules. In Figure 4 it is shown the collision management and the messages exchange between vehicles, LRTMs and HRTM. These procedures may help to improve the capability of the system to face with this kind of event. In particular, the event management is composed of several steps. In this scenario two vehicles generate a collision events, suddenly vehicle generates a beacon message to inform RSU device to spread this event in the area. Moreover, this message is sent towards the LRTM responsible of this area that starts to manage the events by updating the status of the lanes involved by the collisions. In particular, in Figure 4a only the LRTM system is involved in the management of the collision because its severity is low. In the scenario shown in Figure 4b local traffic management was not sufficient to solve traffic congestion. In this case, the HRTM can take global decision to reduce congestion in the area of collisions blocking or limit incoming flow from surrounding areas managed by different LRTMs. Also the message is propagated towards its neighbor LRTMs by using the INTHLI and towards HRTM by exploiting the IHLI. LRTMs provide to spread information about collision and LRTM decision in their area of interest. HRTM will take under consideration this message for the next map update process.

#### 3.2.1. Message Definition

**Figure 5:**
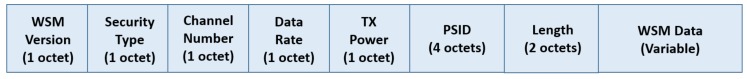
The Format of a WSMP Packet.

In order to implement the proposed idea in a real system, some additional messages need to be introduced. The most important messages are those to manage neighbor list of each vehicle and those concerning the warning messages in order to inform other nodes of something that is happening in the roads. A WAVE specific protocol called WSMP has been developed to carry messages through nodes. The WSMP allows the applications to directly control the lower-layer parameters such as transmit power, data rate, channel number and receiver MAC addresses. The primary motivation for using the WSMP is to reduce the overload. A WSMP packet is shown in Figure 5.

Its overhead is 11 bytes, compared to a minimum of 52 bytes of a UDP/IPv6 packet. If a device receives a WSMP packet that has a *WAVE Short Message* (WSM) Version number not supported by the device, the received WSMP packet will be discarded. The Security Type identifies if the packet is Unsecure, Signed or Encrypted. The Channel Number, Data Rate and TX Power allow the WSMP to directly control the radio parameters. The purpose of the *Provider Service ID* (PSID) field consists in the similar role as the port number of the UDP/TCP packet, i.e., to identify the application that will process the WSM Data. The Length field indicates the number of bytes in the WSM Date, which might have been security-protected as specified in IEEE1609.2 [40].

#### 3.2.2. Main Protocol Messages

On the basis of WSMP protocol, in this work, broadcast and geocast message policies are introduced to improve cooperation between vehicles and to enhance drivers safety. These messages are locally and globally distributed depending on their goals. In particular, when a danger is rising it is important to disseminate information directly to the closest vehicles to notice them about the event. In this way, preventive actions may be done by drivers and vehicles in a shorter time. If the severity of event is high then it is possible to involve other layers to process the event. The warning dissemination into two separate steps allows to avoid indiscriminate flooding on the network reducing packets number and saving resources.

#### 3.2.3. Low Layer Protocol Messages

**Figure 6:**
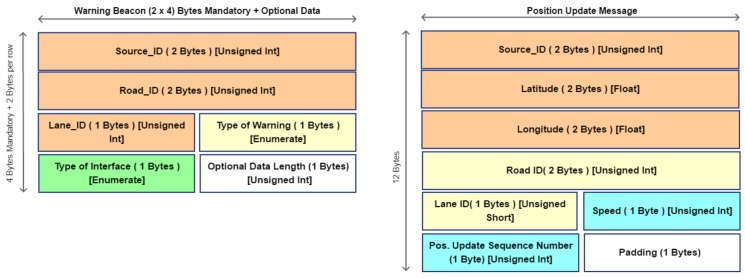
Beacon Low Layer Message Data Structure.

**Figure 7:**
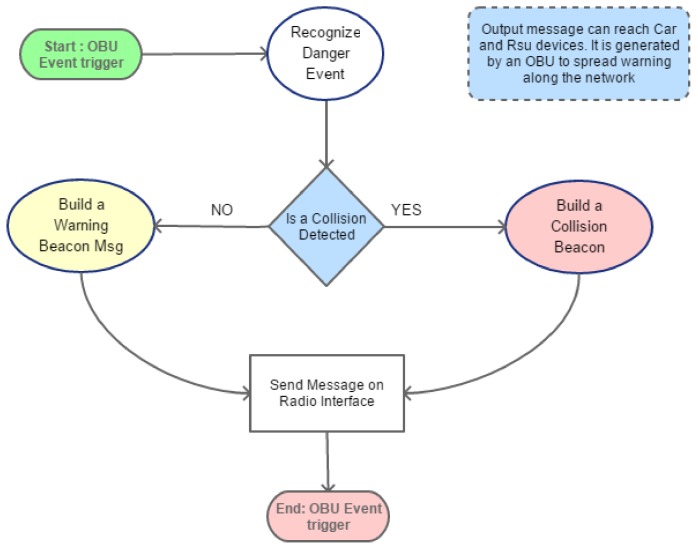
Warning message creation by E-OBU.

On the basis of WSMP protocol, in this work, some messages are introduced to allow cooperation between on-board devices. These messages are introduced for warning advertisement goal and to enhance the pleasure of driving. Collision or high traffic may be the main reasons of congestion. ITS system should be smarter enough to find or forecast a situation like that and realize an opportune policy: it should solve or avoid congestion. Commonly the solution is to close the roads near the involved area but this solution is only temporary because it moves the problem to another area. In fact, there is a high probability that roads will be overloaded. A static solution or pre-programmed policies are not the best choice because they do not take into account the dynamics of the traffic and how the vehicles are moving in the areas. For these reasons, it is important to make a real time monitoring of traffic by acquiring continuously data from vehicles, sensors and devices. It is easy to understand that the amount of data to collect is huge and under this point of view a scalable architecture may help the overall system to enhance the performance. As regards the lower layer, a customized protocol, based on VANET architecture, is able to recognize the events and collect data. The high layer, instead, uses protocol to exchange data about event and to organize data elaboration by distributing tasks among local and global processes. For the low layer, the on-board system collects data by vehicle sensors. These data are locally analyzed for recognizing dangerous events and then they are sent towards the higher layers by exploiting interfaces ILLI and IHLI. These messages are herein reported:

- *Register Message*: this message is sent by vehicle towards RSUs once it changes Road_ID. Once it is received by RSU, it registers the vehicle_ID in the reachable car list if and only if the vehicle is passing though a managed Road_Id. RSU sends back a Register Acknowledgment that contains data about managed road; The RSU discards register message if vehicle that has generated message is not passing on managed Road_Id.
- *POSition Update* (POSUP): each node manages its own neighbor list by receiving the periodical POSUP message. It is sent by each vehicle once the position and current speed are acquired by inner sensors. Destination nodes are the neighbors of vehicle and lead RSU. Its dissemination is bounded to one hop and it is composed as shown in Figure 6. When a POSUP message is received by a RSU, it elaborates vehicle position and places it in the related lane queue of the road. In Table 1 it is possible to view its fields specification.
- *Warning Message*: It is sent by vehicles in the network when something happens during their journey. For example, when a critical situation is recognized by E-OBU by elaborating sensor data. Here two types of warning messages have been considered: *Warning Beacon* (WB) and *Collision Beacon* (CB). Let us describe in detail how these messages are composed. In Figure 6 the WB message is depicted. Beacon is encapsulated in the WSM Data field of WSM packet. Its length is 8 bytes composed of mandatory fields plus other optional rows of 2 bytes length. Their number is signaled in a specified mandatory field (see Table 2). The vehicle elaborates dangerous events as shown in Figure 7.

Vehicles know the road_ID because of communication with RSU. IDs are exchanged by RSU and OBU when vehicle is registered by a RSU. Each RSU manages a set of roads that have a unique ID defined in the ITS.

#### 3.2.4. High Layer Protocol Messages

**Figure 8:**
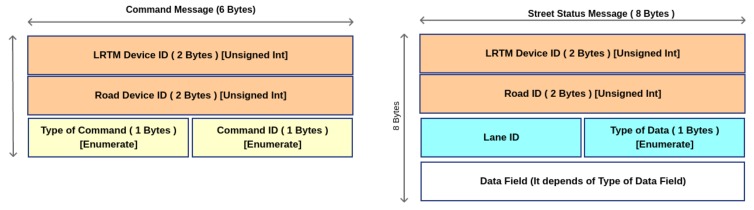
High Layer messages sent on *Inter-Low-Layer Interface* (ILLI) and *Inter-High-Layer Interface* (IHLI).

**Figure 9:**
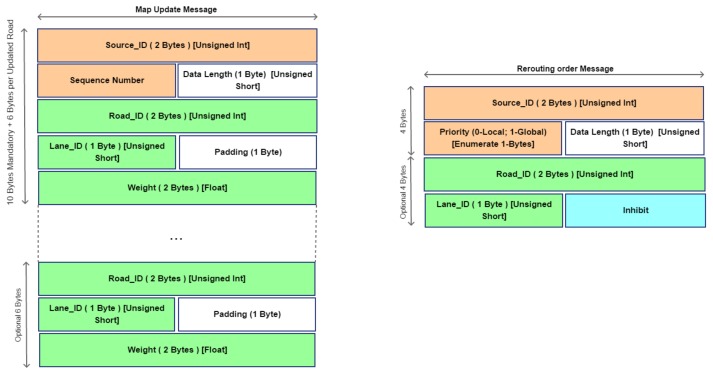
*Map Update Message* (MapUpMsg) and *Rerouting Order Message* (ROMsg) messages format.

In this section, the messages used by the higher layer of the architecture are proposed. Lower layers have the main goal to collect data from vehicles/environments and locally manage traffic in case of recognized events that may congest traffic. Higher layer, instead, has the main goal to keep under control traffic condition globally. In order to allow this kind of task opportune messages have to be sent along the network for keeping updated data on real traffic status. Here the description of the messages is reported:

- *Hello Message* (HM): it is sent between LRTMs and HRTM when a LRTM node starts to work. In order to keep updated the list of active LRTM, this message is sent periodically by exploiting the IHLI interfaces;
- The *Command Message* (CmdMsg): it is one of the most important messages and its main goal is to inform road smart device to make an actuation such as activate a gate control to deny or allow access in a controlled area or by acting on city street lights to change their timing and so on. It is composed as shown in Figure 8. It is sent towards the IHLI and ILLI interfaces as shown in Figure 2; This message is generated by HRTM and LRTM to actuate traffic policies.
- The *Collision Beacon* (CB): it is utilized by vehicles to inform LRTM about a happened collision. Moreover, the LRTMs uses this messages to inform one-hop LRTMs about this event. This message is then forwarded by LRTMs to covered vehicles as Warning Message as already described in Figure 6. *BC* message is composed of two main fields: *RSU_ID* (the ID of the RSU device) and *Lane_ID* (the ID of the lane segment);
- *Street Status Message* (StrStsMsg): it is sent between LRTMs and HRTM. This message is used for updating the status of the covered roads and to bring up data to HRTM. *Low Road Traffic Manager* (LRTM) collects information from lower layers and aggregates data. Periodically these data are reported to the higher layer to update roads status globally at the end of an observation window. The message is composed as shown in Figure 8. Here *Type of Data Field* can be Inhibit or Update. In case of Inhibit message data fields shall not be taken into consideration, otherwise, if the value is set to Update then data fields shall be composed of six bytes that means three float values. The first value represents the weight of the edge, which is called *w*, the second one shall be the density value called π and the last one shall be the traveling delay in minutes called δ. These values will be evaluated by RSU locally as shown in Section 5.3.
  Table 3 specifies the contents of message. It reports three important values used to perform ITS choices in terms of traffic management. In particular, these parameters are referred to a specific lane of a specific road. The road_ID field gives us information about the vertexes of the edge to be considered, instead, the lane_ID field gives us details about the order to recall the right edge. In fact, commonly the edge $e_{i,j}$ may be different of $e_{j,i}$. Thus, the weight of lane in the global maps is represented by the weight of the edge $e_{i,j}$ called $w_{i,j}$ as well as the density factory called $\pi_{i,j}$ and delay term called $\delta_{i,j}$.
- *Map Update Message* (MapUpMsg): it is sent by HRTM to LRTMs periodically along the ILLI interface to allow LRTM to keep updated the weights of the covered roads. In this way vehicles can update their map to perform rerouting actions when triggered. More details are given in Figure 9. In this message, the data length field indicates the number of road information that message contains. The weight coefficient field, instead, reports the new weight coefficient for the related lane identified by the Road_ID and Lane_ID fields.
- *Rerouting Order Message* (ROMsg): This message is generated by LRTMs. It sends ROMsgs through the LLI interface reaching RSUs. RSUs spread this message to vehicles. In order to limit flooding and saving resources, this message has two kinds of priority Global and Local. In case of Global Priority the message has been generated because of HRTM decisions. In this case, all vehicles will receive this message. In case of Local Priority only a limited set of vehicles will receive the message. This last set is composed of vehicles covered by LRTM area of interest. Message will change weights coefficient for some edges to follow LRTM or HRTM decisions. Once received, interested vehicles will evaluate an alternative path to reach their destinations. More details about message format are given in Figure 9. Here is reported the message format. In particular, on the basis of the value of the Priority field is possible to infer the information that message brings up. If the priority field is set to Global this means that the ROMsg is sent by HRTM to actuate a global rerouting. If the priority field, instead, is sent by LRTM this means that is valid only in the area of interest of the LRTM and the rerouting is made only by vehicles that are traveling in this area. Moreover, the content of the message includes also local policies made by LRTM about lanes interested by decision making policies.

#### 3.2.5. Neighbor Vehicles for Short Range Advertisements

In order to avoid an uncontrolled flooding along the network it is important to create a neighbors list for each vehicle to inform them in case of recognized dangers along their journey. Thus, we can define a *l*-th vehicular node equipped with an E-OBU by calling it $n_{l}$. In the same way, the global set of vehicles that are traveling across the ITS managed area is called *N*. In this way we can define $n_{l}\in N$. Each lane is identified in the system as an edge of the ITS graph and it is marked as $e_{i,j}$ where *i* and *j* are the vertexes of the edge ant it belongs to the set of roads called *E*, therefore $e_{i,j}\in E$. A vehicle node $n_{m}$ is a neighbor of $n_{l}$ if their distance falls below a given threshold called $T_{cov}$. In VANETs, the average coverage radius of a generic vehicle belongs to the interval [200, 400] m, and it depends of several parameters, as referred in [43,44]. In this paper, we are not analyzing the effects of the coverage range of IEEE802.11p and DSRC, so we simply consider that $n_{l}$ and $n_{m}$ are neighbors if their Euclidean distance is less than $T_{cov}$. We are also considering a 2D environment (assuming that the considered geographical region belongs to a plane distribution). Each node manages its own neighbor list by receiving the periodical POSUP message sent by each vehicle once the position and current speed are achieved by GPS.

## 4. Vehicle On-Board Equipment

In this sub-section a description (in terms of hardware) of several solutions for *On-Board Unit* (OBU) and *Extended On-Board Unit* (E-OBU) devices is given.

### 4.1. On-Board Unit (OBU)

OBU consists of the control unit, the communication subsystem and the sensor network. Control and communication unit is based on the single board industrial PC of *Embedded Platform for Industrial Computing* (EPIC) form. Type of EPIC module is NANO-9452 and it is a product of IEI Technology Corp., Taiwan. The *Controller Area Network* (CAN) interface is interconnected with EPIC module by internal PC/104+ bus and it creates interface between OBU and sensor network. The sensors represent all those devices used for measuring/sensing of temperature, gas concentration, pressure, vehicle inclination and glass break. These sensors have the task of monitoring status of dangerous and, then, sending detected data to the monitoring centre in order to take the opportune countermeasures to rescue system in case of damage or accident [45]. In [46] the authors describe a device able to connect vehicles to RSUs: the *Single Board Computer* (SBC), based on a prototype of the Institute for Infocomm Research in Singapore. The SBC has the capability to host a CAN interface, while WLAN-based communications have been enabled by means of an installed wireless chip, supporting the IEEE802.11b standard. The SBC device represents an interesting solution for OBUs, also because it adopts some interfaces provided by the *Communications Access for Land Mobiles* (CALM) architecture [47]; the main problem is represented by the absence of an integrated component approach, since each sub-system is interconnected in different boards.

### 4.2. Extended On-Board Unit (E-OBU)

The E-OBU permits to connect on-board sensors of vehicle to VANET services. In this paper, we introduced two types of sensors that, interfaced with the OBU, can increase the on-board safety of passengers. The sensors are the GPS module for geolocalization services and the proximity sensors, used to scan surrounding area of vehicles and raise alarms when an obstacle is found. This on-board device allows to enhance safety of the car and nearest vehicles too, by exchanging warning messages in case of dangerous events. An ad-hoc protocol has been designed to allow communication between nearest vehicles by tackling flooding and link congestions. The E-OBU may represent an improvement of VANET environment that can supply additional information for ITS tasks.

**Figure 10:**
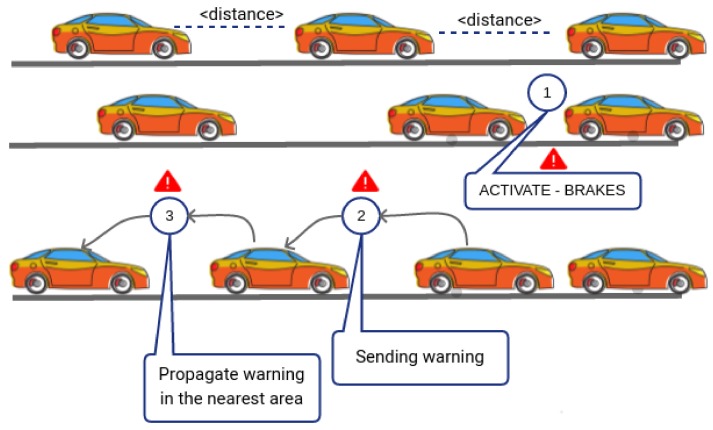
Example of warning event recognized by E-OBUs. Warning message dissemination between neighbor vehicles.

In this work, the on-board sensors are used to scan the surrounding area of the vehicle to avoid undesired collisions commonly recognized as pile-up: they happen because of drivers distractions or in case of a driver with an altered state. When multiple collisions happen, the involved lane suddenly meets congestion due to the obstacles represented by vehicles. If vehicles are equipped with the E-OBU, the proposed idea provides two main actions, triggered by the device. As the first step, when the distance between vehicles is reducing too quickly, then the system acts on brakes in a preventive way to force car braking and, in this way, driver may react braking car by itself. The resultant effect is a reduction of reaction time. The second step is to notify a possible dangerous event to vehicles that are arriving. This case is shown in Figure 10.

**Figure 11:**
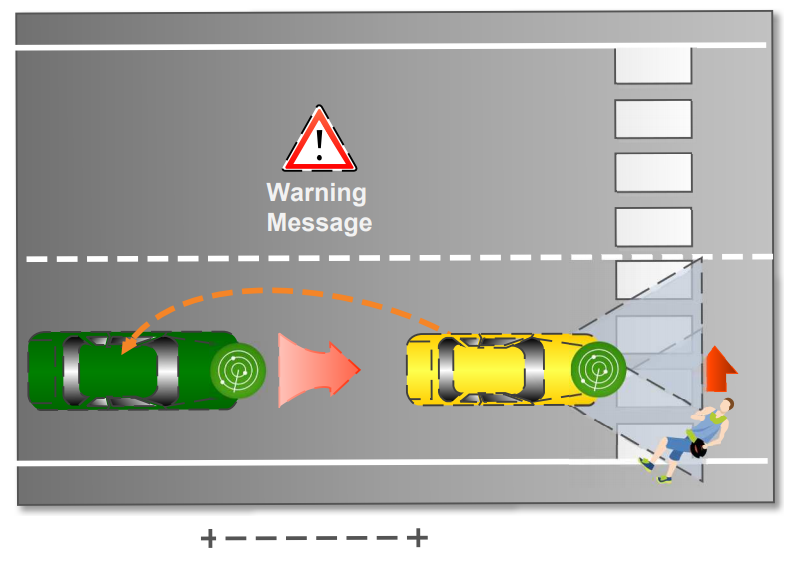
Example of sensors working in the case of a pedestrian starting to cross the road when a car is passing.

Moreover, on-board sensors may help to advice drivers and other vehicles in case of people that are crossing the roads when a car is arriving. Several studies have pointed out this kind of event by raising up attention on safety of pedestrians. An example is shown in Figure 11, where a pedestrian is starting to cross the lane while a car is passing through. The E-OBU recognizes the pedestrian and performs two actions. The first one is alerting the driver about the pedestrian with on-board signaling system and the second one is to send a protocol warning message to the nearest vehicles. This should act on the collision probability by reducing drivers response time.

## 5. Distributed ITS Tasks

ITS tasks are distributed among HRTM and LRTM. In this section the main idea focuses on the possibility to exploit data collected by vehicles enhancing the capability of ITS to provide traffic solution as fast as possible through the distribution of tasks along the defined layers. Fog computing [38] may help to reduce system response time analyzing data gathered from field sensors, speeding up the decision making processes. In this work, data are provided by vehicles, RSUs and traffic sensors. These data are utilized to keep updated roads status. First of all it is necessary to identify main tasks that are involved in the decision making processes.

### 5.1. Road Management

In this sub-section we introduce the way the considered geographical area has been modeled in order to apply the proposed idea. The aim of proposing a novel road model is out of the scope of this paper, so we refer to many works in literature [48,49,50] in which the following approach has been introduced.

The road network is modeled as a weighted oriented graph, G=<V,E>, where *V* is the set of vertexes, ||V||=1,…,c and *E* is the set of edges, ||E||=1,…,m.

**Figure 12:**
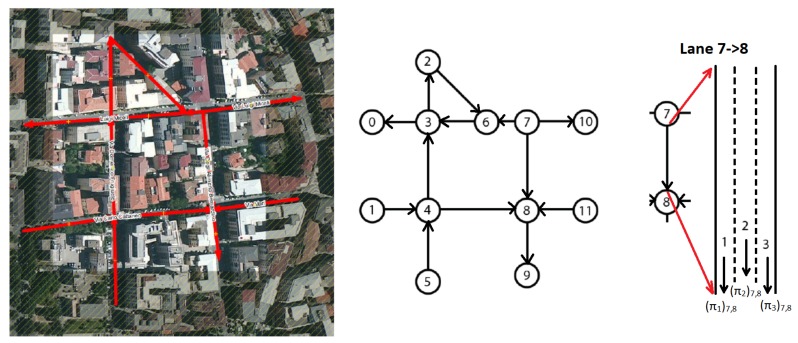
An example of the way a graph can be associated to a geographical map with a set of roads. To be noted that the graph is unidirected, $e_{i,j}\neq e_{j,i}$.

Each edge e∈E represents a lane segment between two vertexes and it has an associated weight w∈W the number of vehicles on the lane, each vertex v∈V represents a cross roads or two lane junction. Figure 12 illustrates the considered model. To be noted that the graph is unidirected, $e_{i,j}\neq e_{j,i}$.

Based on the definition and studies in [51] a density term can be defined as follows:

(1) $${\left(\pi_{k}\right)}_{i,j}=\frac{{\left(w_{k}\right)}_{i,j}}{length(e_{i,j})}$$

(2) $$\Pi_{i,j}=\sum_{k=1}^{N_{i,j}}{\left(\pi_{k}\right)}_{i,j}$$

where $\pi_{k_{i,j}}$ is the traffic density of the *k*-th sub-lane of the lane segment $e_{i,j}$ (see Figure 12 Lane 7->8), defined as the ratio of $w_{k_{i,j}}$ (that is the weight of the *k*-th sub-lane of the lane segment $e_{i,j}$) and the length $l_{i,j}$ of the lane $e_{i,j}$, while $\Pi_{u,z}$ is the average density for the edge *u*, *z* and $N_{i,j}$ is the number of considered lane segments (i.e., in Figure 12 it is possible to note that a road between node 7 and node 8 is composed of 3 lanes) with $k=1,\ldots ,N_{i,j}$. It is necessary to introduce *k* in order to take into account the possibility to have more lanes with same direction in a same road. In particular, from [52,53] it can be written that for a motorway the capacity $c_{i,j}$ of the lane $e_{i,j}$ is:

(3) $$c_{i,j}=C_{i,j}\cdot FHW_{i,j}\cdot FW_{i,j}\cdot FP_{i,j}$$

where $C_{i,j}$ is the ideal capacity, $FHW_{i,j}$ is related to the probability of having heavy vehicles, $FW_{i,j}$ is a factor related to the width of $e_{i,j}$, and $FP_{i,j}$ is a factor related to the driver population. The criterion for choosing the best path is now illustrated. The algorithm (based on *Shortest Path First* (SPF)) is based on reducing the instantaneous road density on all segments and, consequently, the time spent on the road segments. The best path is chosen by:

(4) $$BEST\_path_{k}\left(D\right)=\arg\min_{k}\left\{MCARS\left(path_{k}\left(D\right)\right)\right\}$$

where $path_{k}\left(D\right)$ is the *k*-th available path to reach the destination *D*. The MCARS term represents the considered metric. Considering the lane $e_{i,j}$ belonging to the $path_{k}\left(D\right)$, it is defined as:

(5) $$MCARS_{i,j}=\alpha \cdot \left[\frac{w_{i,j}/length\left(e_{i,j}\right)}{max\_\pi \_path(D)}\right]+\left(1-\alpha \right)\cdot \left[\frac{length\left(e_{i,j}\right)/{\bar{\nu }}_{i,j}}{max\_delay\_path(D)}\right]$$

where α is a weight term (for giving more emphasis to density or to travel delay), max_π_path(D) and max_delay_path(D) are the maximum density and delay terms over all paths to destinations and ${\bar{\nu }}_{i,j}$ is the average speed on lane $e_{i,j}$. Given a path(D) composed by a sequence of segments, the total $MCARS[path_{k}\left(D\right)]$ is just the sum of the each $MCARS_{i,j}$ associated to each segment of path(D).

### 5.2. Road Monitoring

Road monitoring is an activity made by LRTMs. It is made by exploiting data gathering by low layer devices such as E-OBU, RSU and road smart devices. Data are extracted from protocol messages (already described in previous sections). In details, it is possible to achieve data in a real-time way about roads status by elaborating POSUPs and Warnings messages. From POSUP messages it is easy to retrieve the number of vehicles per road or lane and it is possible to understand the density of flows as well as road load status by measuring the lane density as shown in Equation (1). Warning messages, instead, can be used to understand if something is happening on a particular lane or road by analyzing the types of warning. With these data, it will be possible to react to dynamic events by actuating local policies for tackling congestions and collision side effects on traffic. Taking into account the POSUP message the LRTM can evaluate the following measures:

- $w_{i,j}\left(t\right)$: from latitude and longitude it is possible to evaluate the road_ID by simply applying the following steps. First convert latitude and longitude in the two dimensional plane. Once its achieved we can check if the point P(x,y) belongs to a given edge. Edges represent lanes’ road. We have to recall that it is possible to have normal roads with both directions available and one way roads. In this last case, for a right assignment of lane_ID we have to receive at least two POSUP messages for being able to evaluate vehicle direction. However, each lane is bounded by two vertexes that represent a cross roads or two segments junction as shown in Figure 12. Once the lane_ID is found we can update number of vehicles that are on the lane at the time *t*. In other words, $w_{i,j}$ is time dependent, but it is evaluated periodically: this means that $w_{i,j}$ represents a sample in a time interval of length *t*.
- $\pi_{i,j}\left(t\right)$: it is evaluated after the measure of $w_{i,j}$ and represents the density metric of the lane_ID $e_{i,j}$. The higher is the value of density metric the higher is the probability to have congestion on the road and the higher is the time needed to pass through the lane.
- $\delta_{i,j}$: it is the time measured for passing through the lane $e_{i,j}$. It is given by the average value measured by vehicle that changes lanes in the time interval *t*. When the vehicle enters in a lane LRTM devices store the entering time in the local database.

### 5.3. Data Management

**Figure 13:**
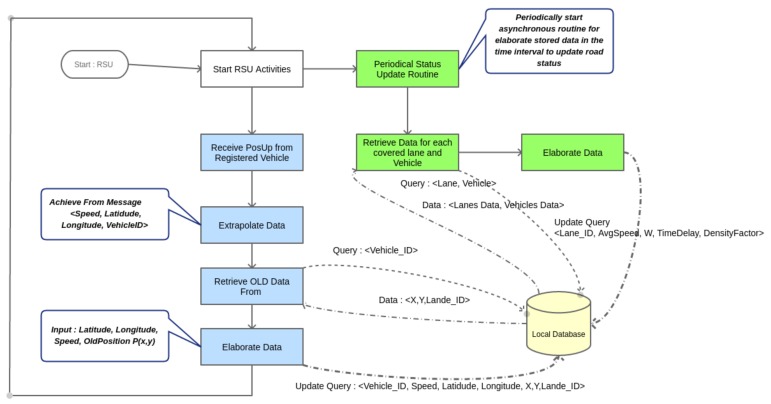
RSU routines performed to keep update road and vehicle variables.

Data collected by low layers is continuously analyzed by LRTM devices. Moreover, it is clear that local policies may help to react quickly to dynamic event that could happen but only local action may not be enough to find a good solution for ITS. Therefore, if the severity of the event is high, its effect may influence surrounding areas and other vehicles can be involved. For solving these side effects, also higher layer can be involved in ITS decision making process by informing it about road conditions. It is clear that in this way the HRTM will be involved only when the severity of blocks is really high. Therefore, HRTM will process a lower number of events. This will reduce the overall computational load and the number of messages that ITS will receive from VANET layer. Of course it is also important that the main map has to be kept updated in terms of road weights. As defined in the previous section, each road has a related weight that represents its status. Once a vehicle enters in the monitored area it downloads the updated maps and performs a routing algorithm to find the better path to reach its destination. In Figure 13 the data elaboration process is performed locally for each RSU. Data are available for LRTMs that can aggregate these data to further update the HRTM by using the *Street Status Message* (StrStsMsg) along the IHLI link. This update is triggered if some road status is altered because of collisions or due to a sensible increasing of traveling delay on that road. However, this message is sent periodically by LRTM with the goal of keeping update the global map status of ITS. Once StrStsMsg is received by HRTM it updates global map. The updated Map will be available for next MapUpMsg sent by HRTM to LRTMs. It is important to recall that HRTM may change weights because of traffic rearrangement to better distribute traffic along the area map. Moreover, some lanes may result inhibited by HRTM because of high traffic level in surrounding areas. This decision making routines can be supplied only at the higher level of architecture because of its global view.

## 6. Performance Evaluation

In this section, the proposed architecture is evaluated in terms of scalability and capability to tackle emergencies such as car collisions and traffic congestions. Moreover, the introduction of E-OBU is evaluated by checking the number of car collisions and their severity in the system.

### 6.1. Simulations Environment

In order to perform our simulations, we designed a custom simulator using the OMNet++ framework [54] building ad-hoc modules for the architecture implementation. Protocol messages and interfaces have been implemented in compound modules written in c++ language by extending *Veins* and *Inet* module. In particular, Inet framework has been utilized for creating the edge/fog layers and cloud services. These custom modules permit to realize the LRTM and HRTM modules, with the related communication interfaces. Vehicle environment has been realized by using the *Veins* module [55] that implements the IEEE802.11p/WAVE protocol and DSRC model. For creating the mobility scenarios we connect SUMO framework [56] with *Veins*. Reference maps have been obtained from real road maps by importing City area from Open Street Maps service. They permit to realize real traffic scenario adopting dynamic behavior. Maps have been populated by choosing sources and sink nodes in the outer region of the maps. Table 4 shows the simulation parameters used in the proposed architecture. In the next section we considered the following parameters:

- *Number of Collision*: it represents the number of collisions due to an accident event in the map;
- *Average Speed*: it is the average speed of the car during its travelling in the map;
- *Travelling Time*: it is the time spent by a car during its travelling;
- *Average Distance*: it is the average distance travelled by car during its path in the map;
- *Number of Packets*: it represents the total number of protocol messages sent during the simulation.

### 6.2. Extended Sensing Evaluation

**Figure 14:**
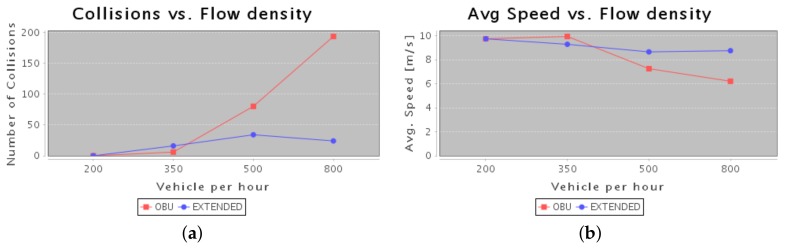
(**a**) Collision number in urban area; (**b**) Average speed in urban area.

In this section, the performances of E-OBU are reported. In particular, in Figure 14a the trend of collisions encountered in the monitored area is shown. In case of vehicles equipped with the E-OBU, there are more collected information about surrounding area. In case of suddenly direction/speed change it is possible to avoid pileup if warning messages between nearest vehicles are exchanged; this trend is depicted in Figure 14a as well.

In Figure 14b the average vehicles’ speed trend is depicted. The higher is the density of the cars in the area the higher is the probability to have collisions. This affects the average speed that decreases because of the road congestion.

**Figure 15:**
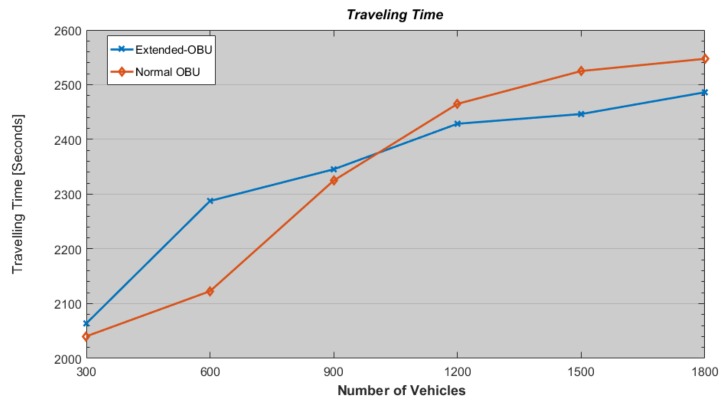
Travelling Time versus Flow Density.

Regarding the Traveling time experienced by vehicles (due to the higher number of collisions and because of rerouting strategy), vehicles that are not equipped with E-OBU measure a higher traveling time as shown in Figure 15. In particular, with a lower number of vehicles in the considered area, in case of E-OBU vehicles measure a higher traveling time because their average speed decrease due to alerting strategies. Once the number of vehicles increases over a certain value, then the higher number of collisions influences the whole area and vehicles experience a higher traveling time.

### 6.3. Proposed Architecture Evaluation

Our proposal is compared with the common protocol used in VANET environments for signaling warning to others vehicles and another protocol based on cloud services where ITS runs its tasks to reassign traffic load around the covered area. We have to point out some considerations that have driven our simulations. First of all, collisions between vehicles may happen in the closest areas around the cross-road junctions. We considered the E-OBU able to rise warning when an obstacle is found to the vehicle.

To have comparable results between our proposal and other ones, the simulated vehicles are equipped in other environment with the same OBU. As consequence, the number of protocol messages increased, but it permits to reduce the number of pileups. In the first campaign, we propose system behavior versus the increasing of vehicle density in the monitored area, taking as fixed parameters the ones shown in Table 4.

**Figure 16:**
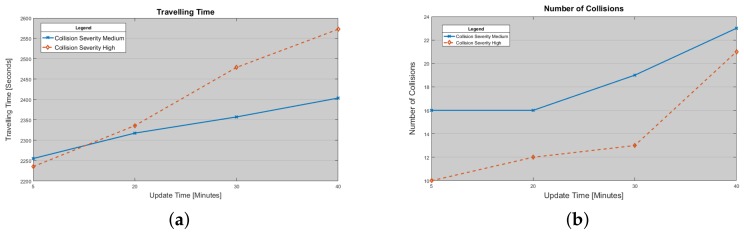
(**a**) Average Traveling Time experienced by vehicle vs. Map Update Time; (**b**) Number of collision versus Map update Time.

First of all, it is important to evaluate how the ITS system installed in the higher architecture layer impacts on traffic conditions. Regarding ITS implementation and its behavior we implemented a solution already proposed in the [57] where the *Ad-hoc On-demand Distance Vector* (AODV) protocol is used to disseminate ITS messages in the VANET environment. Therefore, when the ITS term is reported, we refer to the proposed model in [57]. In Figure 16a, the trend of average time that a vehicle may spend in the city is depicted. In order to perform this simulation campaign we consider two kinds of collision. The first one is called medium severity. Here the average duration of a collision is 10 min. In the second case, called high severity, the average collision duration is 20 min. It is shown that, increasing the updating time related to the map weights, the traveling time increases. This means that ITS manager reacts more slowly with the increasing of update time. In this scenario, we evaluate the capability of the system to respond at collision events. In this figure it has been demonstrated that the higher is the collision severity in terms of duration the higher is the traveling time experienced by vehicle. This happen because system redistributes traffic density too slowly and it involves more areas. In this way, it is clear that ITS solutions only based on centralized services may not be enough to face congestion issues. In Figure 16b, the number of collisions are reported: it is possible to note that the increasing of update interval, in the ITS core, increases vehicle density in certain area. This issue augments the probability that a collision could happen. In case of high severity the number of measured collisions are lesser than medium severity because it has been measured that the average speed is lesser than average speed measured in the case of medium severity. This happens because of vehicle queues that are longer and numerous.

**Figure 17:**
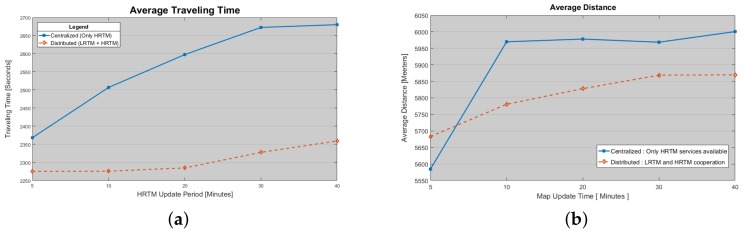
(**a**) Average Traveling time spent by vehicles in the city map area. Here Centralized vs. Decentralized ITS behaviors are depicted; (**b**) Average Traveled Distance measured by vehicles.

In this last scenario the ITS centralized approach is compared with the distributed and multi-layer approaches presented in this paper. In Figure 17a the trend of traveling time (that represents the time spent by a vehicle in the city area covered by ITS service) is represented. It has been demonstrated that, in the case of the distributed approach, it is possible to reduce the queue waiting time by approaching faster to traffic issues, such as collisions or congestions. This allows system to respond faster to these events by rerouting vehicles and avoiding local congestions.

In Figure 17b the average traveled distance per vehicle is depicted for centralized and decentralized ITS solutions. Here it is shown that the decentralized approach allows vehicles to travel a shorter distance than the centralized ITS, because of the possibility to find an alternate path when something is recognized by local infrastructure. In the case of centralized solution, instead, vehicle rerouting can be performed only after the complete updating of the map, with the current road status. Moreover, it is also possible to observe that the centralized solution is very sensible to the choice of update interval. With the increasing of the update interval, an increasing of the severity of the traffic jams in the surrounding areas has been observed. The result is the increasing of the traveled distance because of higher density factor per involved lane.

**Figure 18:**
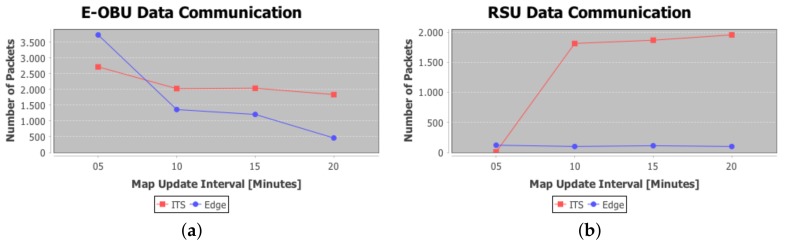
(**a**) *Extended On-Board Unit* (E-OBU) Protocol data messages related to dangerous events; (**b**) RSU Protocol data messages involved in warning dissemination and rerouting activities.

In Figure 18a it is shown the protocol messages trend related to warning dissemination. Here, the better behavior of decentralized architecture able to distribute ITS tasks is proved. In particular, with the increasing of Map Update time interval a lesser number of messages are pushed into the network for both approaches, but it is also shown that global driver comfort is drastically reduced for the centralized approach; this is is also shown in Figure 17. Moreover, because of increasing congestion severity in the involved roads, the probability to realize collisions or congestion increases too. In the distributed approach, instead, the system can reduce the congestion effects, keeping under control the presence of vehicles on the lanes and reducing the probability to have collisions between vehicles. This may help to reduce congestions and traffic jams as well as pileups. Moreover, it is also shown that the higher number of messages for E-OBU in the distributed environments is measured due to the messages of map update sent by HRTM to the lower layer. In Figure 18b it is shown the trend of messages exchanged between RSU . The number of packets sent in the centralized environment is higher than the distributed one because of the dissemination of map update and the higher number of warnings that the vehicle layer generates. In fact, the higher is the density of vehicles in a specific area, the higher is the probability to recognize a dangerous event. In this work it has been demonstrated how a decentralized architecture can help to reduce the number of messages needed for managing ITS, keeping high performances and driver comfort indexes, such as traveling time and traveled distances.

## 7. Conclusions

In this work, a new distributed architecture is proposed to improve ITS management for enhancing the performance of traffic solutions. To this aim, an E-OBU is proposed by exploiting VANET infrastructure to gather and disseminate information with the main goal to increase safety with a better management of road traffic. The E-OBU is able to recognize dangerous events and notify alerts to neighbor vehicles. The spreading of these messages permits to reduce collision number and probability of road blocks. The full integration of on-board devices such as GPS, proximity sensors and communication network infrastructure has permitted to enhance also the driving comfort. Under the point of view of ITS management, the main idea is to spread information collected by vehicles in the high architecture layers, distributing the elaboration tasks between local and global devices. In particular, the work shows how it is possible to attribute global policies and decision to HRTM to perform rerouting policies to balance vehicle density among lanes to mitigate congestion and reduce the experienced traveling time. On the other side the LRTM can perform local policies to take quick decision for tackling collisions or traffic jams events. Roads map has been modeled on the basis of a graph structure, where cross-roads represent the vertexes of the graph and lanes are its edges. Moreover, a protocol has been proposed to allow a more reliable and efficient data dissemination between layers. Finally simulative campaigns have been carried out to demonstrate the goodness of proposal: an E-OBU can enhance the safety level of drivers and may help to reduce the number of collisions. Moreover, with a safer environments it is possible to improve driver comfort increasing the average speed of vehicle and reducing the traveling time because of a lower number of collisions and forced re-routing. The achieved results have demonstrated that the proposed protocol that exploits the VANET infrastructure for vehicle communication can enhance several quality indexes along the roads, by allowing the spreading of important data in beaconing messages, based on the IEEE802.11p and DSRC communications.

## Figures and Tables

**Table 1:** *POSition Update* (POSUP) Message fields specification.

Field Name	Type of Data	Length in Byte	Description
*Source_ID*	Unsigned int	2	ID of E-OBU that generates the message
*Latitude*	Float	2	Latitude of Vehicle
*Longitude*	Float	2	Longitude of Vehicle
*Road ID*	Unsigned Int	2	Road ID
*Lane ID*	Unsigned Short	1	Lane ID
*Speed*	Unsigned Short	1	Speed in Kmph
*Sequence Number*	Unsigned Short	1	Sequence Number
*Padding*	Unsigned Short	1	N.A.

**Table 2:** Warning Message fields specification.

Field Name	Type of Data	Length in Byte	Description
*Source_ID*	Unsigned int	2	ID of E-OBU or RSU that generates the message
*Road_ID*	Unsigned int	2	unique ID that represents the road globally in the ITS
*Lane_ID*	Unsigned short	1	ID of road lane involved in warning
*TOW*	Enumerate	1	Warning or Collision Beacon
*Interface origin*	Enumerate	1	[ILLI, HLI, ...]
*Optional Data Length*	Unsigned Short	1	Optional Data length in Byte

**Table 3:** *Street Status Message* (StrStsMsg) : Message fields specification.

Field Name	Type of Data	Length in Byte	Description	
*LRTM_ID*	Unsigned int	2	ID of LRTM device	
*Road_ID*	Unsigned int	2	ID that represents the road globally in the ITS	
*Lane_ID*	Unsigned short	1	ID of road lane involved in update status	
*Type of Data*	Enumerate	1	0-Inhibit	1-Update
*Weight data field*	Float	2	new lane metric value: $w_{i,j}$	
*Density Factory*	Float	2	Density factory: $\pi_{i,j}$	
*Traveling Delay*	Float	2	Delay value: $\delta_{i,j}$	

**Table 4:** Simulation parameters.

Param. Name	Param. Value	Description
City Area size	11 Km × 12 Km	City map area size managed by ITS
Number of FLows	4–6	Regulate vehicle density in the monitored area
Flow 1	50–300	From 8 a.m. to 12 p.m.: *Vehicle per Hour* (Vph)
Flow 2	50–300	From 8 a.m. to 2 p.m.: *Vehicle per Hour* (Vph)
Flow 3	50–300	From 8 p.m. to 3 p.m.: *Vehicle per Hour* (Vph)
Flow 4	50–300	From 8 p.m. to 6 p.m.: *Vehicle per Hour* (Vph)
Flow 5	50–300	From 14 p.m. to 7 p.m.: *Vehicle per Hour* (Vph)
Flow 6	50–300	From 14 p.m. to 7 p.m.: *Vehicle per Hour* (Vph)
Simulation time	From 0 a.m. to 8 p.m.	Time of the day take into consideration for sims
RSUs coverage	100–300 m	Coverage radius area for RSU devices
Vehicle warning propagation	80–300 m	coverage distance for flooding warning
RSU dissemination	1–16 Hops	RSU multi-hop warning dissemination
E-OBU Sensing Distance	3–15 m	Sensors coverage distance
Tx Power for Vanet Device	20 dBm	
Wave lenght	0.051	5.0 GHz of frequency (IEEE802.11p)
Fading Factor	2	fading factor in the vehicular environment
Rx Antenna Gain	2	Rx antenna gain
Tx Antenna Gain	2	Tx antenna gain
Antenna Height of vehicle	1.50 m	Vehicular antenna height from the ground
Antenna Height of RSU	3.0 m	Rsu antenna height from the ground
